# A dataset of COVID-19 x-ray chest images

**DOI:** 10.1016/j.dib.2023.109000

**Published:** 2023-02-18

**Authors:** Mohammad Fraiwan, Natheer Khasawneh, Basheer Khassawneh, Ali Ibnian

**Affiliations:** a Department of Computer Engineering, Jordan University of Science and Technology, P.O. Box 3030, Irbid 22110, Jordan; b Department of Software Engineering, Jordan University of Science and Technology, Jordan; c Department of Internal Medicine, Jordan University of Science and Technology, Jordan

**Keywords:** COVID-19, Chest X-ray, Artificial intelligence, Diagnosis, Detection, Deep learning

## Abstract

The distinction between normal chest x-ray (CXR) images and abnormal ones containing features of disease (e.g., opacities, consolidation, etc.) is important for accurate medical diagnosis. CXR images contain valuable information concerning the physiological and pathological state of the lungs and airways. In addition, they provide information about the heart, chest bones, and some arteries (e.g., Aorta and pulmonary arteries). Deep learning artificial intelligence has taken great strides in the development of sophisticated medical models in a wide range of applications. More specifically, it has been shown to provide highly accurate diagnosis and detection tools. The dataset presented in this article contains the chest x-ray images from the examination of confirmed COVID-19 subjects, who were admitted for a multiday stay at a local hospital in northern Jordan. To provide a diverse dataset, only one CXR image per subject was included in the data. The dataset can be used for the development of automated methods that detect COVID-19 from CXR images (COVID-19 vs. normal) and distinguish pneumonia caused by COVID-19 from other pulmonary diseases. ©202x The Author(s). Published by Elsevier Inc. This is an open access article under the CC BY-NC-ND license (http://creativecommons.org/licenses/by-nc-nd/4.0/)

**Specifications Table**

**Table:** 

Subject	Biomedical Engineering
Specific subject area	Machine Learning; Pulmonary diseases; COVID-19; diagnosis applications
Type of data	Images (.JPG files)
How data were acquired	The CXR images were reviewed using the MicroDicom viewer version 3.8.1 (see https://www.microdicom.com/), and exported as high resolution images (i.e., 1024 × 851 pixels).
Data format	Raw.
Parameters for data collection	None.
Description of data collection	The images were collected with the aim of identifying lung damage causes by COVID-19 pneumonia. Thus, COVID-19 cases confirmed by real-time reverse transcription-polymerase chain reaction (RT-PCR) were selected. Out of these cases, only subjects requiring hospital stay were chosen. Moreover, the CXR images were chosen to be at least 5 days since the hospital admittance. This will include the most likely CXR images to show lung damage, and exclude mild cases of the disease. After that, all CXR images were inspected by two specialized physicians to confirm the lung involvement.
Data source location	Institution: King Abdullah University Hospital
	City/Town/Region: Ramtha/Irbid
	Country: Jordan
Data accessibility	Repository name: Mendeley Data
	Data identification number: 10.17632/xztwjmktrg.2
	Direct URL to data: https://doi.org/10.17632/xztwjmktrg.2
	Instructions for accessing these data: None.
Related research article	Khasawneh, N.; Fraiwan, M.; Fraiwan, L.;Khassawneh, B.; Ibnian, A. Detection of COVID-19 from Chest X-ray Images Using Deep Convolutional Neural Networks. Sensors 2021, 21, 5940. https://doi.org/10.3390/s21175940

## Value of the Data

• Images in the dataset can be used in image processing and artificial intelligence (AI) research in the area of pulmonary diseases. These data provide high quality CXR images from 368 subjects experiencing confirmed COVID-19 lung complications. The images in the dataset will provide more variety and volume to similar public datasets, which maybe suffering from the scarcity of publicly available COVID-19 CXR images [1], [6].
• Medical professionals and researchers will benefit from these images for designing or testing automated methods for the detection of pulmonary diseases from CXR images. Moreover, the images can be used to educate and train medical students.
• The data can be reused in many ways. First, the image files can be processed to detect and extract features using a multitude of algorithms. Second, new machine learning models for disease diagnosis can be trained and tested. Third, image processing and artificial intelligence methods can be developed for the detection of specific features in the CXR images (e.g., consolidations), see Behzadi-khormouji et al. [2].

## 1 Objective

Chest X-ray images are one of the most valuable and frequently used means to diagnose pulmonary diseases among other forms of chest-related ailments. Recent advances in computational and processing power has enables sophisticated artificial intelligence algorithms that can automate many clinical activities. This in turn can increase the accuracy and speed of solving a lot of medical problems, and reduce operator/clinician errors. In particular, deep learning AI methods have displayed great potential in implementing image-based diagnosis tools that can meet or exceed clinical requirements. To this end, however, very large datasets are required to correctly and reliably train and test the AI models. The dataset described in this manuscript provides valuable chest x-ray images that can enrich, diversify, and expand existing datasets.

## 2 Data Description

The dataset [4] includes CXR images from 368 subjects (215 males, 153 females). The mean subject age was 63.15 years with a standard deviation (SD) of 14.8 and an age range of [31 months, 96 years]. The diagnosis was performed using at least one positive real-time reverse transcription-polymerase chain reaction (RT-PCR) test and the subjects were admitted to the hospital on the recommendation of the specialists at King Abdullah University hospital (KAUH). The hospital stay ranged from 5 days to 6 weeks, with some subjects discharged after negative PCR tests and satisfactory health improvement, and others passed away.

**Fig. 1:**
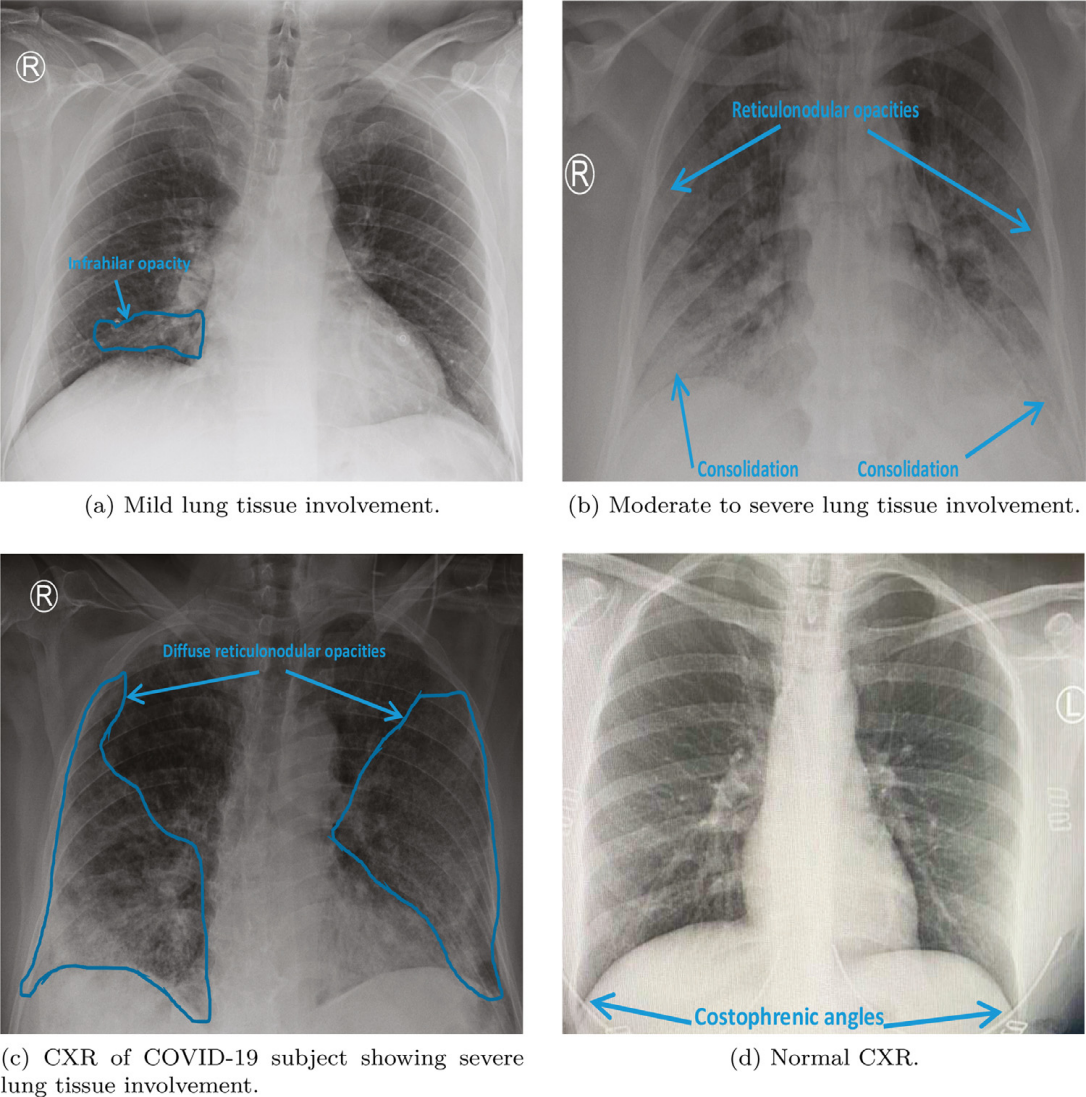
CXR images with various levels of lung tissue invovlement.

The COVID-19 subjects, who displayed/exhibited clinical symptoms, are highly likely to produce an adverse/abnormal lung X-ray profile [8]. Recent studies reported that these CXR images show patchy or diffuse reticular nodular opacities and consolidation, with basal, peripheral and bilateral predominance [3]. For example, Fig. 1a shows the CXR of a mild case of lung tissue involvement with right infrahilar reticular nodular opacity. Moreover, a moderate to severe case of lung tissue involvement with right lower zone lung consolidation and diffuse bilateral airspace reticular-nodular opacities is shown in Fig. 1b. Although, as the CXR shows, they are more prominent on peripheral parts of lower zones. Furthermore, a severe case of lung tissue involvement is shown in Fig. 1c. In this CXR image, diffuse bilateral airspace reticular-nodular opacities are shown to be more prominent on peripheral parts of the lower zones, and ground glass opacity in both lungs predominant in mid and lower zones. On the other hand, Fig. 1d shows an unremarkable CXR with clear lungs and acute costophrenic angles (i.e., normal). The normal image is not part of the published dataset and included here for comparison.

The dataset contains one CXR image per subject. On the other hand, some relevant databases contained multiple CXR images per subject (e.g., one dataset from Spain was published over Twitter and contained 134 CXR images from 50 COVID-19 subjects (see https://twitter.com/ChestImaging)). Moreover, other datasets were automatically compiled from different COVID-19 relevant articles, which may result in great number of duplications [7].

The images in the dataset were compiled from the corresponding patients records viewed using MicroDicom viewer version 3.8.1 (see https://www.microdicom.com/.) As the dataset involved patients with hospital stays spanning multiple days, each patients record contained multiple CXR images (i.e., for most patients one CXR was taken per day). A specialist viewed the entire patients history and chose only one CXR image that best shows the disease effects on the lungs. To preserve anonymity, only the CXR view was cropped from the viewer. The images were numbered in a sequence from 1 to 368. The images have a resolution of 1024×851 pixels.

In foresight, we believe that the data collection should have been performed in a manner that enables much wider avenues for research. Hopefully, future and related works can avoid the following pitfalls:

• The entire history record of each patient should have been preserved along with the disease progression or healing state, and the sequence in time of the image. This would enable research into comparing sequences of CXR images for healing/degeneration. Moreover, this would greatly expand the dataset, albeit it may result in close lookalikes.
• The data shared and described are in.jpg format and not.dicom format, which would be more scientifically acceptable for future processing by other researchers. Those files should have been anonymized and shared.
• Although the resolution of the images is more than what is typically required by deep learning and image processing algorithms (e.g., most well-known models require a resolution in the range of 224× 224 to 331×331) [5], keeping the files in.dicom format would have allowed the flexibility for exporting into higher resolutions in case of future advancements.
• The resulting CXR images were not cropped, which means that part of the image contains black irrelevant parts that should be removed for more accurately representative results.
• Some CXR images reveal medical devices wiring equipment (i.e., patients state does not allow removal of these connections). Such artifacts maybe detected by deep learning algorithms to indicate disease state.

## 3 Experimental Design, Materials and Methods

COVID-19 cases with confirmed diagnosis using PCR tests were admitted to the King Abdullah University Hospital (KAUH). The general treatment protocol requires taking CXR images upon admission and every two days, although this may differ depending on the severity of the case. Hospital electronic records were fetched, which hold the patient full name, date of birth, gender, and date of the radiograph. The CXR records are kept in Digital Imaging and Communications in Medicine (DICOM) format. Only patients with medium to severe cases requiring multiday stay were chosen, and the CXR images after at least 5 days of admittance were picked. The system stores every CXR image that were conducted for the patient. Thus, CXR images with low quality, poor orientation, or showing excessive artifacts (e.g., jewelry, wiring to hospital devices) were excluded. Other CXR images of the same subject were ignored to keep the dataset as diverse as possible. The images were exported to.JPG format with the specified resolution using the software MicroDicom viewer version 3.8.1.

Radiograph markings showing subject/doctor names were removed or redacted. Other markings on the images may include the anatomical side of body (L for Left or R for right), project type (AP for Anterior-Posterior (AP), or PA for Posterior- Anterior), positioning of the body if other than standing (e.g., supine), and the type of X-ray machine if portable. In this dataset, 25 images were taken in supine position, and 39 images were captured using a portable device, and all except one used AP projection. All of these variations happened due to the conditions of the patients, radiologists, equipment, or hospital at the time.

## Ethics Statement

All study participants (the adult subjects, their parents in case of underage subjects, or the authorized person in case of incapacitated adults) provided written informed consent to being included in the study and allowing their data to be shared. This study was conducted according to the guidelines of the Declaration of Helsinki and approved by the institutional review board at King Abdullah University Hospital and Jordan University of Science and Technology, Jordan (Ref. 91/136/2020). The data collection was carried out under the relevant guidelines and regulations. The authors have the right to share the data publicly.

## Declaration of Competing Interest

The authors declare that they have no known conflicts of interest that influenced this study in any form.

## CRediT authorship contribution statement

**Mohammad Fraiwan:** Conceptualization, Software, Validation, Data curation, Writing – original draft, Supervision, Project administration. **Natheer Khasawneh:** Software, Validation, Data curation, Supervision, Project administration. **Basheer Khassawneh:** Methodology, Investigation, Resources, Supervision. **Ali Ibnian:** Validation, Investigation, Data curation.

## Declaration of Competing Interest

The authors declare that they have no known conflicts of interest that influenced this study in any form.

## Data Availability

COVID-19 Chest X-ray Images (Original data) (Mendeley Data).
